# FBR2 modulates ferroptosis via the SIRT3/p53 pathway to ameliorate pulmonary fibrosis

**DOI:** 10.3389/fphar.2025.1509665

**Published:** 2025-02-11

**Authors:** Yu Cheng, Yang Jiao, Wan Wei, Mengjia Kou, Yaodong Cai, Yang Li, Hao Li, Tonghua Liu

**Affiliations:** ^1^ Graduate School, Beijing University of Chinese Medicine, Beijing, China; ^2^ Department of Respiratory, Dongfang Hospital, Beijing University of Chinese Medicine, Beijing, China; ^3^ Department of Respiratory and Critical Care Medicine, Beijing Jiangong Hospital, Beijing, China; ^4^ Key Laboratory of Health Cultivation of the Ministry of Education, Dongfang Hospital, Beijing University of Chinese Medicine, Beijing, China

**Keywords:** pulmonary fibrosis, bleomycin, Chinese medicine, FBR2, ferroptosis, SIRT3/p53 pathway, acetylation

## Abstract

**Background:**

Idiopathic Pulmonary Fibrosis (IPF), an interstitial lung disease of unknown etiology, remains incurable with current therapies, which fail to halt disease progression or restore lung function. However, Feibi Recipe No. 2 (FBR2), a clinically validated traditional Chinese medicine formula, exhibits potential as an IPF treatment.

**Objective:**

This study aimed to investigate the regulatory effect of FBR2 on ferroptosis through the SIRT3/p53 pathway and its therapeutic potential in improving IPF.

**Methods:**

Pulmonary fibrosis was induced in C57BL/6J mice by intratracheal instillation of Bleomycin (BLM), followed by FBR2 treatment via gavage. Assessments encompassed histopathology, ELISA for cytokine detection, IHC and Western blot for protein expression analysis, and qRT-PCR for gene expression quantification. Transmission electron microscopy (TEM) was used to observe mitochondrial morphology. The roles of Erastin and the SIRT3 inhibitor 3-TYP were also explored to elucidate FBR2’s mechanisms of action.

**Results:**

FBR2 treatment significantly mitigated BLM-induced lung injury in mice, as evidenced by improved body weight and survival rates, and reduced levels of inflammatory cytokines, including IL-6 and TNF-α. FBR2 decreased collagen deposition in lung tissue, as shown by Masson’s staining and IHC detection of Col-I and α-SMA, confirming its anti-fibrotic effects. It also reduced iron and MDA levels in lung tissue, increased GSH-Px activity, improved mitochondrial morphology, and enhanced the expression of GPX4 and SLC7A11, indicating its ferroptosis-inhibitory capacity. Furthermore, FBR2 increased SIRT3 levels and suppressed p53 and its acetylated forms, promoting the translocation of p53 from the nucleus to the cytoplasm where it co-localized with SIRT3. The protective effects of FBR2 were reversed by Erastin, confirming the central role of ferroptosis in pulmonary fibrosis treatment. The use of 3-TYP further confirmed FBR2’s intervention in ferroptosis and cellular senescence through the SIRT3/p53 pathway.

**Conclusion:**

FBR2 shows therapeutic potential in a BLM-induced pulmonary fibrosis mouse model, with its effects mediated through modulation of the ferroptosis pathway via the SIRT3/p53 mechanism. This study provides novel evidence for the targeted treatment of IPF and offers further insights into its pathogenesis.

## 1 Introduction

Idiopathic pulmonary fibrosis (IPF) is an interstitial lung disease of unknown etiology, characterized by progressive pulmonary fibrosis. Clinically, it presents as worsening dyspnea, often culminating in respiratory failure and death. The majority of patients are over the age of 65, with the disease’s onset and progression thought to be associated with genetics, environmental exposures (including smoking), and aging (Pardo and Selman, 2016; Hilberg et al., 2022; Ma et al., 2022). The median survival time after IPF diagnosis is approximately 3–5 years, with about 10% of patients experiencing acute exacerbations annually (Homma et al., 2022; Podolanczuk et al., 2023). Besides lung transplantation, a combination of interventions is used to alleviate symptoms and improve lung function and to mitigate the risk of acute exacerbations. These include pharmacological treatments with antifibrotic drugs such as pirfenidone and nintedanib, oxygen therapy, and pulmonary rehabilitation (Liu GabrielleY. et al., 2022). Despite the availability of treatments, none can halt disease progression, provide a cure, or restore lung function, and some treatments are constrained by significant side effects and tolerability issues.

The pathogenesis of IPF centers on the imbalance of apoptosis in alveolar epithelial cells and fibroblasts, encompassing multifaceted disorders such as endoplasmic reticulum stress, telomere length homeostasis, mitochondrial dysfunction, oxidative/antioxidant balance, Th2/Th1 balance, macrophage M1-M2 polarization, and epithelial-mesenchymal transition (EMT), ultimately leading to abnormal accumulation of extracellular matrix and pathological remodeling of lung tissue (Wang et al., 2021; Mei et al., 2022). Despite comprehensive research, the complex etiology of IPF remains poorly understood, and targeted therapies have demonstrated only modest efficacy.

Feibi Recipe No. 2 (FBR2), initially proposed by Master of Traditional Chinese Medicine (TCM), Professor Zhou Ping’an, has been refined and studied by Professor Jiao Yang’s research team. Our team has validated the efficacy of this TCM compound decoction through clinical trials (Fu et al., 2015), as well as *in vitro* (Gu et al., 2022) and *in vivo* (Pang et al., 2022) studies. Our team has previously demonstrated that FBR2 can regulate pro-inflammatory and pro-fibrotic effects in the lungs through pathways such as Nuclear Factor (NF-κB) and Smad2/Smad3 (Wang et al., 2020; Wan et al., 2022); it can balance autophagy/oxidative stress damage through pathways such as Nrf2, PINK1/Parkin, TFEB, and GSK-3β/mTOR (Gu et al., 2022; Liu et al., 2022b; Pang et al., 2022).

Ferroptosis is a novel form of non-apoptotic cell death characterized by iron accumulation-dependent lipid peroxidation and increased levels of reactive oxygen species (ROS). It has been suggested that ferroptosis may play a pivotal role in the pathogenesis and progression of IPF (Cheng et al., 2021). Our preliminary research indicates that the pulmonary fibrosis process in BLM-treated mice is closely associated with a surge in ROS and lipid peroxidation, such as malondialdehyde (MDA), and that FBR2 can ameliorate this phenomenon. However, the specific role of ferroptosis in the therapeutic effects of FBR2 on pulmonary fibrosis is not yet established. Sirtuin 3 (SIRT3), a mitochondrial histone deacetylase, has been recognized as an anti-aging gene, and its dysregulation is implicated in the pathophysiological processes of pulmonary fibrosis (Sosulski et al., 2017). The p53 protein, upon exposure to various cellular stresses, is upregulated and acetylated, thereby participating in the transcriptional regulation of processes such as apoptosis, cellular senescence, and ferroptosis (Wei et al., 2020). Research indicates that SIRT3 can inhibit cellular senescence and ferroptosis by reducing p53 acetylation levels (Chen et al., 2021; Li et al., 2023). Nevertheless, the involvement of the SIRT3/p53 pathway in the therapeutic effects of FBR2 on BLM-induced pulmonary fibrosis remains to be elucidated. This study aims to explore the role of ferroptosis and the upstream molecular regulatory mechanisms in the treatment of BLM-induced pulmonary fibrosis by FBR2 *in vivo* experiment.

## 2 Materials and methods

### 2.1 Reagents and antibodies

Hydrochloric acid bleomycin (BLM, 420770) was purchased from Nippon Kayaku (Takasaki, Japan). 3-TYP (HY-108331) and Erastin (HY-15763) were sourced from MedChem Express (MCE, New Jersey, United States). Antibodies including Collagen I (Col-I; A1352), Solute Carrier Family 7 Member 11 (SLC7A11/xCT; A2413), Glutathione Peroxidase 4 (GPX4; A11243), and SlRT3 (A5718) were all purchased from Abclonal. The nti-p21 antibody (ab188224) was obtained from Abcam. Alpha-smooth muscle actin (α-SMA, 80008-1-AP) and p53 monoclonal antibodies (60283-2-Ig) were purchased from Proteintech. The acetyl-p53 (Lys379) antibody (2570S) was purchased from Cell Signaling Technology. The β-Actin antibody (AF7018) was sourced from Affinity. Lastly, the horseradish peroxidase (HRP)-labelled polyclonal goat anti-rabbit IgG (MD912565) was sourced from MDL, while the deacetylase inhibitor mixture (P1112) was acquired from Beyotime Biotechnology (Shanghai, China).

### 2.2 Preparation of FBR2

The FBR2 prescription comprises six botanical drugs: *Astragalus mongholicus*, *Rhodiola crenulata*, *Lonicera japonica*, *Scutellaria baicalensis*, *Salvia miltiorrhiza*, and *Glycyrrhiza uralensis* (the botanical names have been verified with Medicinal Plant Names Services (MPNS) (http://mpns.kew.org) database as of 1 September 2024), in a ratio of 3:3:3:2:2:1 (for specific details, refer to Supplementary Table 1). In this study, the dosage form is a granule provided by Beijing Tcmages Pharmaceutical Co., Ltd. The daily dose of FBR2 formula granules for adults is 54.43 g, equivalent to 140 g of crude botanical drugs. Based on the human-animal dose conversion standard, a dose of 8.17 g/kg of FBR2 granules was determined for oral gavage in mice (Wei et al., 2010). FBR2 granules are prepared at a concentration of 0.82 g/mL in double-distilled water for *in vivo* experiments (Pang et al., 2022; Liu et al., 2022c) The granules are prepared by mixing individual botanical drug granules according to the dosage, with the botanical drug/extract equivalence ratio and specific dosage detailed in Supplementary Table 2. The manufacturing process of Chinese medicinal granules includes decoction, concentration, addition of excipients, drying, and granulation. Each botanical drug granule is identified and quality-controlled according to the “National Medical Products Administration Standards for National Medical Products.” The test results are presented in Supplementary Table 3 and the raw dataset.

### 2.3 Ultra-high-pressure liquid chromatography (UHPLC-MS/MS) analysis of FBR2 sample

The analytical method for metabolite detection was provided by Qingdao STD Standard Testing Co., Ltd. Sample preparation involved homogenizing 100 mg of sample, adding 1 mL of 70% methanol, and disrupting with an automatic grinder. After centrifugation at 12,000 rpm for 10 min at 4°C, the supernatant was diluted and filtered through a 0.22 μm PTFE filter. The chromatographic separation was performed on a Thermo Vanquish UHPLC system using a Zorbax Eclipse C18 column (1.8 μm, 2.1 × 100 mm). The mobile phase consisted of 0.1% formic acid (A) and acetonitrile (B) with a flow rate of 0.3 mL/min. The gradient elution and column conditions are detailed in Supplementary Table 4. Mass spectrometric analysis was conducted on a Q-Exactive HF mass spectrometer in both positive and negative modes, scanning from m/z 100 to 1500. Key parameters included a heater temperature of 325°C, sheath gas flow at 45 arb, and electrospray voltage at 3.5 kV. Post-acquisition analysis was streamlined using Compound Discoverer 3.3 for peak alignment and extraction. Compound identification relied on secondary MS data matched against Thermo mzCloud and mzValut databases for precise characterization.

### 2.4 Animal models and protocols

The animal experimental procedures of this study were reviewed and approved by the Animal Ethics Experimentation Committee of Beijing University of Chinese Medicine (BUCM) (Ethics number: BUCM-2023102003-4053). Eight-week-old male C57BL/6J mice, weighing 20–22 g, were obtained from Beijing Vital River Experimental Animal Technology Co., Ltd. and housed in the SPF barrier facility at the BUCM’s Animal Experiment Center, where they were maintained under conditions consistent with previous studies. (Liu GabrielleY. et al., 2022). After a 1-week acclimatization period, mice were administered intratracheally with either bleomycin (2.5 mg/kg, 50 µL) or an equivalent volume of saline on day 0 to establish the pulmonary fibrosis model, following our established protocol and confirmed dosages (Zhang et al., 2024).

The experimental mice were allocated into groups: Sham, BLM, FBR2, Erastin (Era), and 3-TYP, with 10 mice per group (Figure 2A). Interventions were initiated from day 1 to day 20 post-modeling. FBR2 or an equivalent volume of saline was administered daily via gavage. Erastin (20 mg/kg) (Huo et al., 2016; Wang et al., 2022) and 3-TYP (20 mg/kg) (Kuang et al., 2023) or equivalent volumes of DMSO solvent were administered via intraperitoneal injection on alternate days, with dosages adjusted based on preliminary experiments. Tissue collection was performed on the 21st day.

### 2.5 Histological study

Lung tissues were fixed in 4% paraformaldehyde at 4°C for 24 h and processed for paraffin embedding and 4 µm sectioning. Sections were stained with Hematoxylin and Eosin (H&E), Masson’s trichrome, and Perl’s Prussian blue (PPB) for histological evaluation. β-galactosidase staining on 10 μm OCT-embedded cryosections was performed to assess cellular senescence. The severity of alveolitis and pulmonary fibrosis in lung tissues was scored using the systems developed by Szapiel et al. (1979) and Ashcroft et al. (1988). Immunohistochemical (IHC) was performed using primary antibodies against α-SMA (1: 200 dilution) and Col-I (1: 50 dilution), followed by HRP-conjugated secondary antibodies and visualized with DAB. For immunofluorescence (IF), sections were treated with antibodies against p53 (1: 200 dilution) and SIRT3 (1: 100 dilution), then labeled with corresponding secondary antibodies and counterstained with DAPI for nuclei visualization. All specimens were examined and acquired using a Leica fluorescence microscope. Image analysis was performed using ImageJ and optimized with Photoshop, with strict standardization to ensure consistency across all samples.

### 2.6 Total iron, MDA and GSH-Px assay

Biochemical analyses of lung tissue samples were conducted to determine levels of total iron, MDA, and glutathione peroxidase (GSH-Px). For total iron measurement using the Total Iron Colorimetric Assay Kit (E-BC-K772-M, Elabscience, Wuhan, China) at 593 nm, lung tissue was homogenized in ice-cold PBS at 1:9 (w/v) and centrifuged at 12,000 × g for 10 min at 4°C, with the supernatant collected for analysis following the kit instructions. MDA levels were quantified by the MDA Assay Kit (S0131S, Beyotime, Shanghai, China) at 535 nm, based on the MDA-TBA reaction. Here, lung tissue was homogenized in PBS at 1:10 (w/v), centrifuged at 12,000 × g for 10 min at 4°C, and the MDA content determined per the kit protocol. GSH-Px activity was assessed with the Glutathione Peroxidase Assay Kit (A005-1-2, Nanjing Jiancheng Bioengineering Institute, Nanjing, China) at 412 nm. Lung tissue was homogenized in 50 mM Tris-HCl buffer (pH 7.4) at 1:9 (w/v), centrifuged at 3,000 × g for 10 min at 4°C, and the supernatant used for the assay as described in the kit manual. All procedures strictly followed the manufacturers’ protocols to ensure the accuracy and reproducibility of the results.

### 2.7 ELISA

Enzyme-Linked Immunosorbent Assay (ELISA) was utilized for quantifying Tumor Necrosis Factor-α (TNF-α) and Interleukin-6 (IL-6) in mouse serum. The assays were performed with the JL10484 and JL20268 ELISA kits from Jonln Technologies (Shanghai, China), strictly following the manufacturer’s protocols. The methodology included the use of pre-coated assay plates and a series of incubations with standards, samples, biotinylated antibodies, and HRP-conjugated streptavidin, culminating in colorimetric measurement at 450 nm.

### 2.8 Transmission electron microscopy

Lung tissues were fixed with 2.5% glutaraldehyde and post-fixed with 1% osmium tetroxide. Following dehydration through an ethanol series, the samples were embedded in epoxy resin, sectioned into 60–80 nm ultrathin slices, and subjected to uranyl acetate and lead citrate double staining. The ultrastructure was examined using a transmission electron microscope (Hitachi; H-7650) at an accelerating voltage of 80.0 kV, captured at ×30,000 magnification.

### 2.9 Western blot

Western blot (WB) was conducted according to conventional protocols. Briefly, protein from pulmonary tissues was extracted with Cell lysis buffer (Beyotime, cat. P0013, China). Proteins were loaded in SDS-PAGE gels and transferred to a nitrocellulose (NC) membrane (GVS, cat. 1215458, United States). The membrane was then blocked and incubated with the primary antibodies overnight at 4°C, followed by incubation with secondary antibodies for 1 h at room temperature. Except for β-Actin, which was diluted at a ratio of 1:3000, all other primary antibodies were diluted at 1:1000. Membranes were exposed and developed after immersion in ECL reagent mixture using a chemiluminescence imaging system (ChemiScope6100, CLINX, China). The expression levels of the proteins of interest were analyzed and quantified relative to the internal reference protein β-Actin. All determinations were performed independently and repeated at least three times.

### 2.10 qRT-PCR

Total RNA was extracted from frozen lung tissues using TRIzol reagent (Aidlab, RN0102), followed by reverse transcription to cDNA with the SuperScript III kit (EXONGEN, A502). The quantitative real-time PCR (qRT-PCR) was performed on a Real-Time PCR System (Longgene, Q2000B) using Sybr qPCR mix (ABI-invitrogen, 4472920). The primer sequences utilized in the qRT-PCR analysis are presented in Table 1. The cycling conditions were optimized as follows: 95°C for 5 min, followed by 40 cycles of 95°C for 10 s, 58°C for 20 s, and 72°C for 20 s. The amplification efficiency and specificity were confirmed by analyzing the melting curves. Data were normalized to Actin and analyzed using the 2^−ΔΔCt^ method.

**TABLE 1:** Sequences of primers used for qRT-PCR analysis.

Gene	Forward primer sequence	Reverse primer sequence
Actin	CTC​CTG​AGC​GCA​AGT​ACT​CT	TAC​TCC​TGC​TTG​CTG​ATC​CAC
SLC7A11	GGC​ACC​GTC​ATC​GGA​TCA​G	CTC​CAC​AGG​CAG​ACC​AGA​AAA
P21	CCT​GGT​GAT​GTC​CGA​CCT​G	CCA​TGA​GCG​CAT​CGC​AAT​C
SIRT3	GAG​CGG​CCT​CTA​CAG​CAA​C	GGA​AGT​AGT​GAG​TGA​CAT​TGG​G

### 2.11 Statistical analysis

Data analysis was performed using GraphPad Prism (V9.0), and graphical representations were generated accordingly. Results are expressed as the mean ± standard deviation (SD). Statistical comparisons among groups were conducted using one-way analysis of variance (ANOVA), followed by Tukey’s *post hoc* test for multiple comparisons. A *P*-value ≤0.05 was considered statistically significant.

## 3 Results

### 3.1 Identification of metabolites in FBR2

**FIGURE 1:**
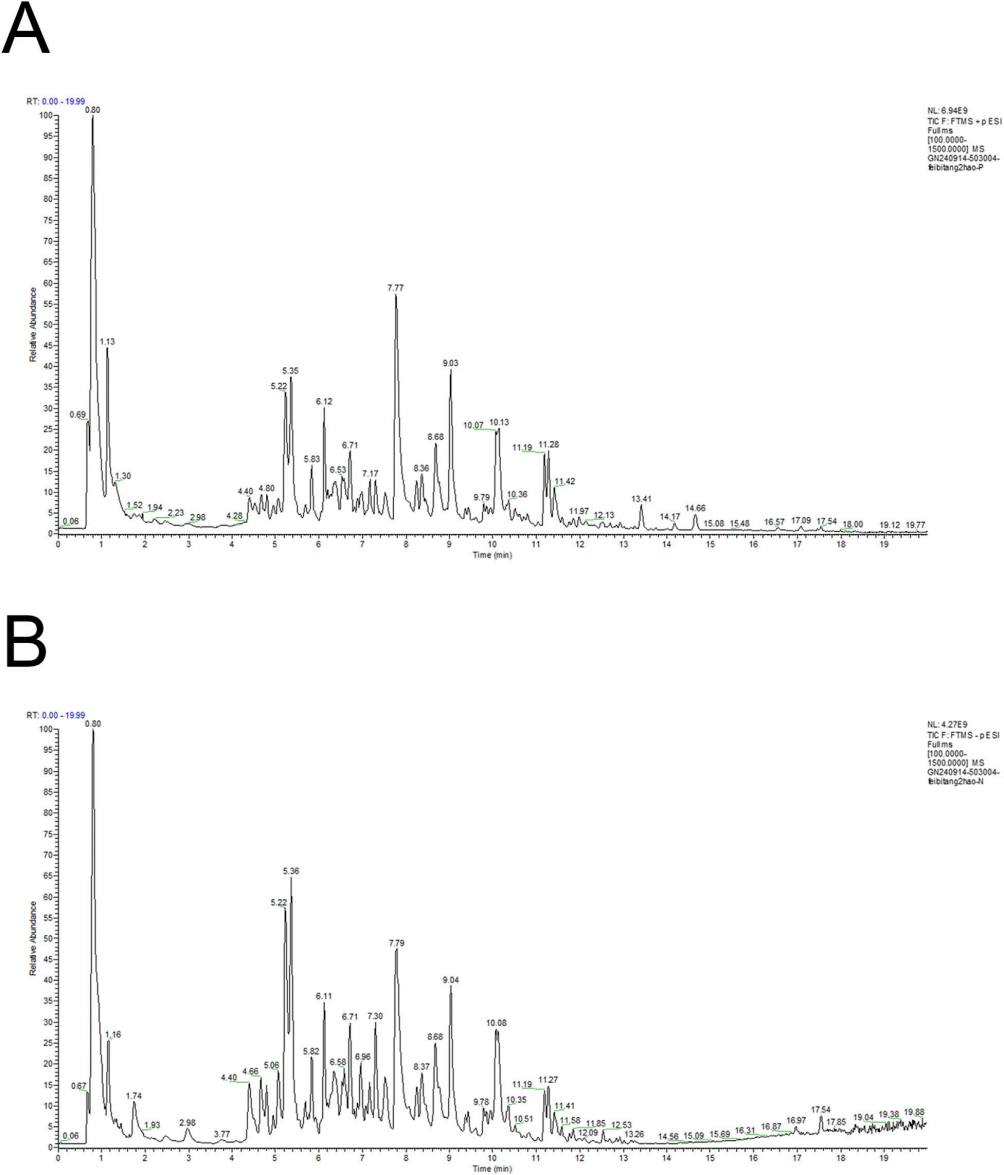
Total ion chromatogram of FBR2 in positive ion mode **(A)** and negative ion mode **(B)**.

The total ion current (TIC) chromatogram of FBR2 is depicted in Figure 1. A comprehensive list of the metabolites identified in FBR2, along with their molecular details, is presented in Supplementary Table 5. The identified metabolites in FBR2 can be categorized into several classes, including flavonoids, terpenoids, organic acids, and others, reflecting the rich chemical diversity of the sample. Our group previously screened and validated certain metabolites in treating pulmonary fibrosis, such as Quercetin, Calycosin, Astragaloside IV, and Polydatin (Tong et al., 2021; Liu et al., 2022b; Li et al., 2024). The most abundant compounds in FBR2, as indicated by their relative concentration in micrograms per milliliter, include Baicalin (953.17 μg/mL), Wogonoside (391.86 μg/mL), Wogonin (303.63 μg/mL), Oren-gedin A (187.81 μg/mL), and Chlorogenic acid (159.42 μg/mL). Other representative components include 7-hydroxycoumarin, Caffeic acid, Calycosin, Ononin, Cantharidin and Cryptotanshinone. These compounds represent the primary constituents of FBR2 and are likely contributors to its pharmacological effects.

### 3.2 FBR2 ameliorates pulmonary injury in BLM-induced mice

**FIGURE 2:**
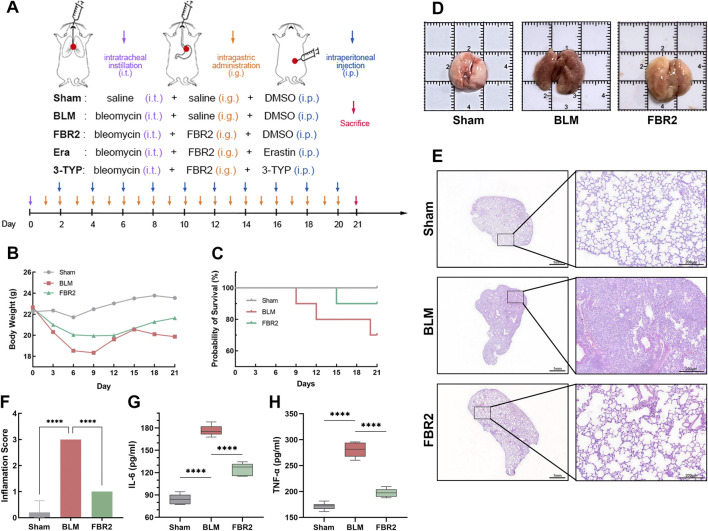
FBR2 ameliorates pulmonary injury in BLM-induced mice. **(A)** Schematic of experimental groups and intervention protocol. **(B, C)** Mouse body weight changes and Kaplan-Meier survival curves. (n = 10) **(D)** Representative gross anatomical views of mouse lungs, harvested after thorough cardiac perfusion. The side length of each square grid is 1 cm. **(E)** Representative full scans and magnified images of H&E staining. scale bars represent 1 mm and 200 μm, respectively. **(F)** Quantitative analysis of alveolitis status in H&E-stained sections according to the Szapiel criteria. (n = 5) **(G, H)** Expression of TNF-α and IL-6 in serum detected by ELISA. (n = 6) ****p < 0.0001.

Statistical analysis revealed that body weight in BLM-treated mice declined rapidly post-modeling compared to the Sham group, whereas those administered FBR2 exhibited a slower decrease, followed by a steady increase after day 12 (Figure 2B). In terms of mortality, no deaths occurred in the Sham group, three deaths occurred in BLM, and one death in FBR2 (Figure 2C). Gross lung anatomical photographs revealed that normal lung tissue, after thorough perfusion and flushing, should resemble the Sham group, showing a light pink color, smooth surface, and rounded, elastic morphology. In contrast, the BLM group displayed darker coloration, with visible dark red reticular patterns, irregular nodules, and congestion; the lung lobes were tough, inelastic, and deformed. Lungs from the FBR2 group appeared yellowish-white, with irregular patches, and a surface that was nearly smooth and elastic (Figure 2D). H&E staining showed that the alveoli in the Sham group were clear, with thin alveolar walls, intact structure without collapse, and no inflammatory cell infiltration. The BLM group exhibited damaged alveolar structures, with large areas of collapse and fusion, thickened interstitium, indicative of fibrosis, and significant inflammatory cell infiltration. The FBR2 group showed significant improvement in interstitial thickening and inflammatory infiltration compared to BLM group (Figure 2E). Quantitative analysis based on Szapel criteria further confirmed that severe alveolitis in the BLM group was significantly improved by FBR2 (Figure 2F). IL-6 and TNF-α, both pro-inflammatory cytokines, can induce inflammation and activate fibroblast proliferation (Bonella et al., 2023). In this study, both IL-6 and TNF-α in the serum of the BLM group were significantly higher than those in the Sham group, while FBR2 significantly improved this situation. In summary, FBR2 can ameliorate the general condition and lung injury in BLM-induced mice.

### 3.3 FBR2 improves pulmonary fibrosis in BLM-induced mice

**FIGURE 3:**
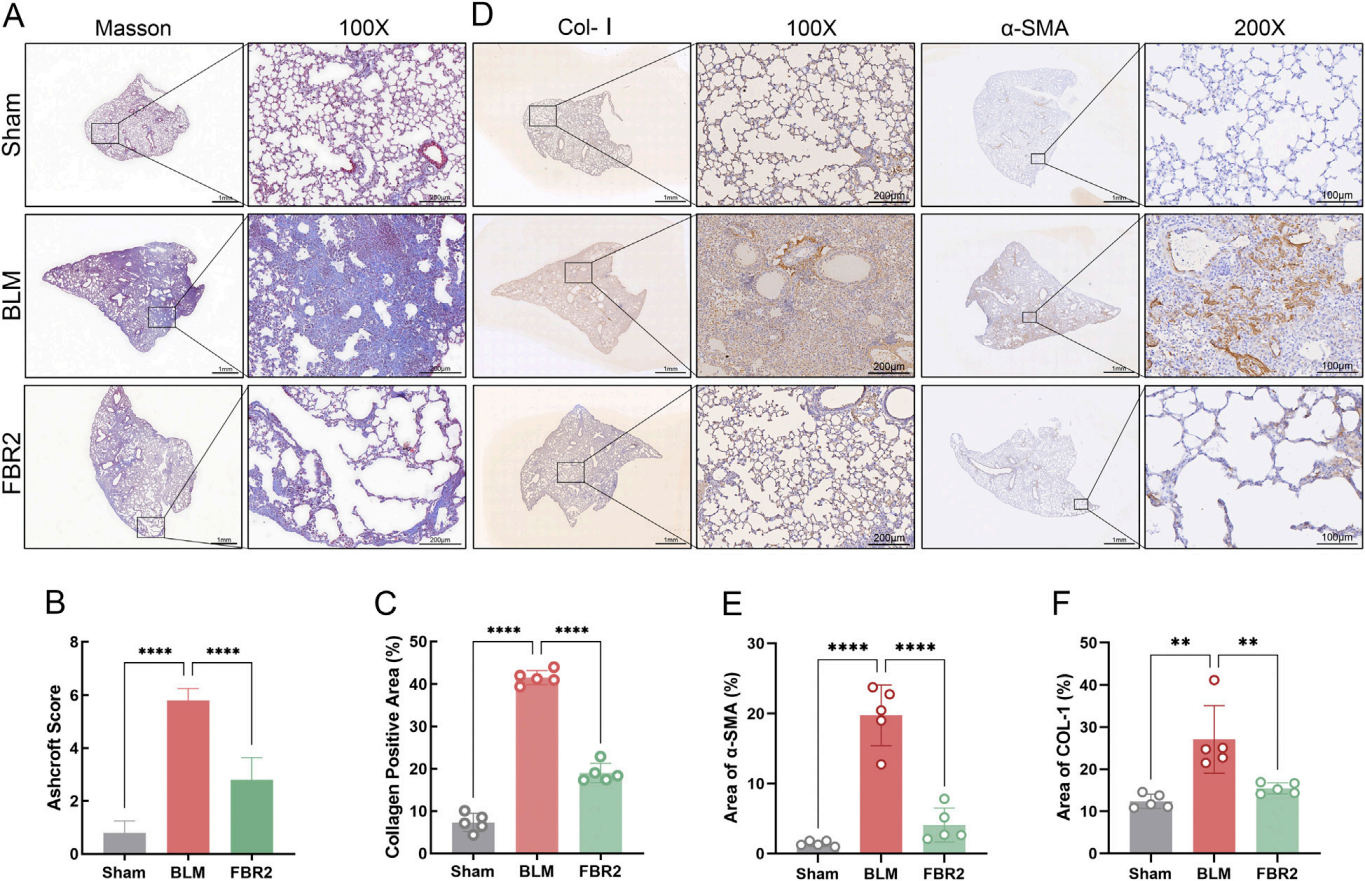
FBR2 improves pulmonary fibrosis in BLM-induced mice. **(A)** Representative full scans and magnified images of Masson’s trichrome staining. Scale bars are 1 mm and 200 μm, respectively. **(B, C)** Quantitative analysis of Masson’s trichrome-stained sections based on the Ashcroft score and collagen area percentage. (n = 5) **(D)** Expression of Col-I and α-SMA in lung tissue detected by IHC. **(E, F)** Quantitative analysis of the percentage area of positive staining for Col-I and α-SMA. (n = 5) **p < 0.01; ****p < 0.0001.

To verify the therapeutic effect of FBR2 on BLM-induced pulmonary fibrosis in mice, Masson staining was conducted, revealing a substantial deposition of collagen fibers in the lung tissue of BLM mice, which was significantly improved in the FBR2 group (Figure 3A). The Ashcroft standard, utilized to evaluate the severity of pulmonary fibrosis, and the quantification of collagen-positive area further substantiated this finding (Figures 3B,C). Col-I, a primary extracellular matrix component, indicates the extent of collagen deposition; α-SMA, a marker of myofibroblast proliferation, reflects the progression of fibrosis. IHC analysis revealed a significant increase in Col-I in the lung tissue of BLM mice, which was mitigated by FBR2. Higher magnification revealed prominent α-SMA staining in fibroblasts, smooth muscle cells, and some macrophages in the BLM group, with a marked decrease in the FBR2 group (Figure 3D). These findings are also supported by quantitative analysis of the positively stained areas (Figures 3E,F). In summary, FBR2 can ameliorate pulmonary fibrosis in BLM-induced mice.

### 3.4 FBR2 inhibits ferroptosis in BLM-induced mice

**FIGURE 4:**
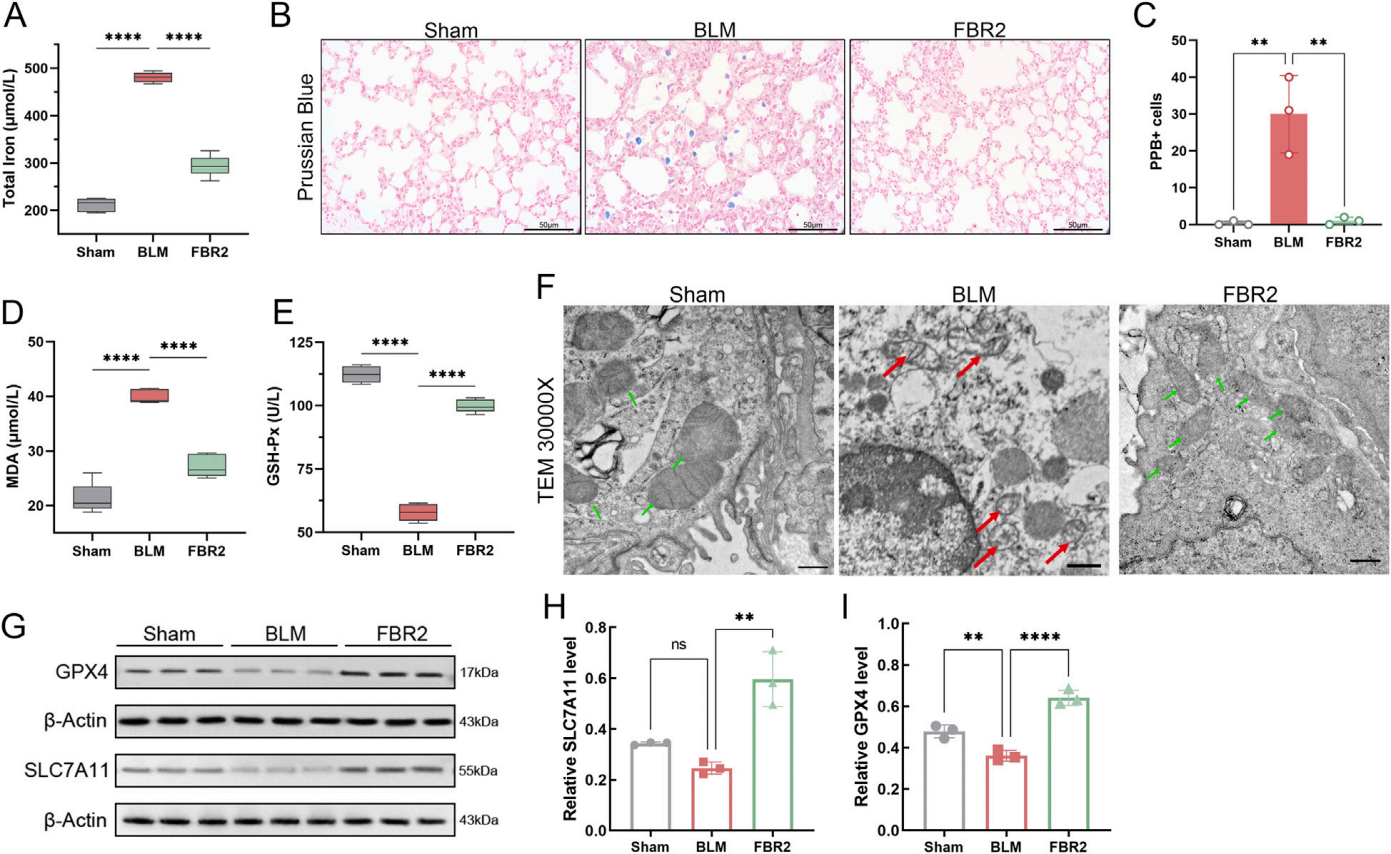
FBR2 inhibits ferroptosis in BLM-induced mice. **(A)** Total iron content in lung tissue. (n = 6) **(B)** Histochemical analysis of iron using PPB staining. Scale bar = 50 μm. **(C)** Quantitative analysis of PPB-positive cell counts. (n = 3) **(D, E)** Biochemical assays for the levels of GSH-Px and MDA in lung tissue. (n = 6) **(F)** Subcellular characteristic images in mouse lung tissue obtained by TEM. Green arrows indicate relatively healthy mitochondria, while red arrows point to abnormal mitochondria, characterized by shrunken morphology, increased membrane density, and reduced cristae. Scale bar = 500 nm. **(G)** Western blot analysis of the ferroptosis-related proteins SLC7A11 and GPX4 in lung tissue. **(H, I)** Densitometric analysis of SLC7A11 and GPX4 protein levels. (n = 3) **p < 0.01; ****p < 0.0001; ns p > 0.05.

Our study investigated the interventional effect of FBR2 on ferroptosis. We first confirmed iron deposition in the lungs of BLM-treated mice through measurement of total iron, PPB staining, and quantification of PPB-positive cells, which were alleviated with FBR2 treatment (Figures 4A–C). MDA, a critical product of lipid peroxidation, and GSH-Px, which reduces cellular oxidative damage by neutralizing lipid peroxides, were significantly accumulated and decreased, respectively, in the BLM group, respectively, with improvements observed in the FBR2 group (Figures 4D,E). TEM revealed that while mitochondria in the Sham group were intact with clear membrane structures, those in the BLM group displayed characteristic signs of ferroptosis, including significant atrophy, increased membrane density, and reduced cristae. In contrast, mitochondria in the FBR2 group showed only mild structural blurring and edema (Figure 4F). WB analysis of ferroptosis defense markers demonstrated that the level of SLC7A11 was lower in the BLM group, albeit not significantly, and further increased in the FBR2 group. The expression of GPX4 was significantly reduced by BLM, while FBR2 promoted its expression (Figures 4G–I). Based on the above findings, it can be concluded that BLM-induced pulmonary fibrosis in mice is associated with ferroptosis changes, and FBR2 can ameliorate cellular ferroptosis.

### 3.5 FBR2 intervenes in the SIRT3/p53 pathway in BLM-induced mice

**FIGURE 5:**
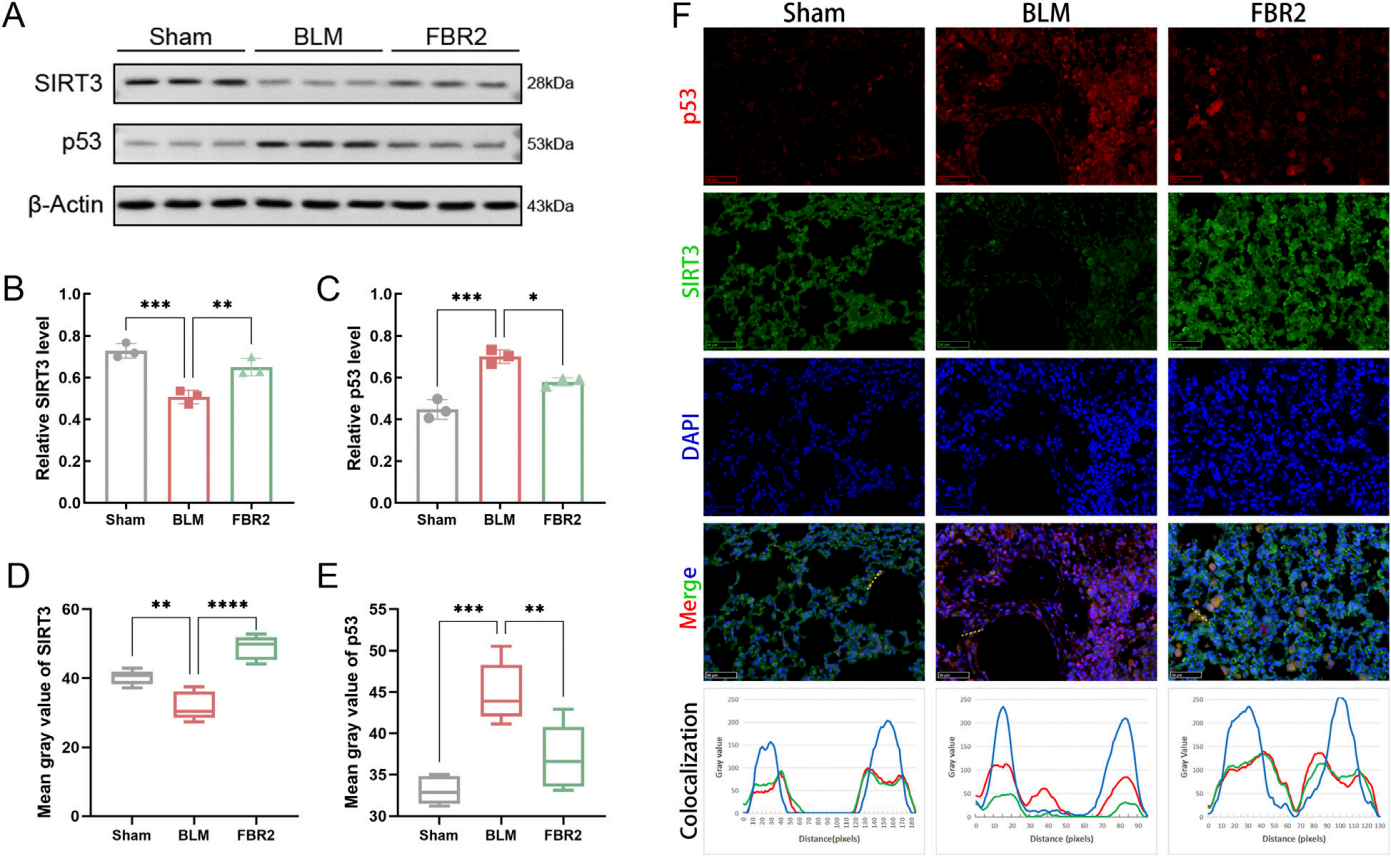
FBR2 intervenes in the SIRT3/p53 pathway in BLM-induced mice. **(A)** Western blot detection of SIRT3 and p53 in lung tissue. **(B, C)** Densitometric analysis of SIRT3 and p53 protein levels. (n = 3) **(D, E)** Quantitative analysis of the mean fluorescence intensity of SIRT3 and p53. (n = 5) **(F)** Immunofluorescence images of mouse lung tissue stained with DAPI (blue), p53 (red), and SIRT3 (green). Scale bar = 50 μm. A yellow dashed line is drawn in the merge image, and a curve graph is constructed with the distance along this line (pixels) as the *x*-axis and the grayscale values of the colors in each channel along the line as the *y*-axis, to reflect the localization of the proteins. *p < 0.05; **p < 0.01; ***p < 0.001.

To elucidate the roles of SIRT3/p53 in the therapeutic effects of FBR2, we initially employed WB analysis to assess their expression levels. SIRT3 expression was significantly diminished by BLM induction but was effectively restored with FBR2 treatment (Figures 5A,B). Conversely, p53 was markedly activated under BLM-induced stress and its upregulation was mitigated by FBR2 (Figures 5A,C). IF imaging corroborated WB results, showing that FBR2 ameliorated the BLM-induced increase in p53 signal and the suppression of SIRT3 in mouse lung tissue (Figures 5D–F). Colocalization analysis revealed that BLM induction enhanced p53 nuclear localization, and FBR2 treatment promoted p53 translocation to the cytoplasm, where it coincided with the peak of SIRT3’s extranuclear intensity, suggesting a potential direct interaction (Figure 5F).

### 3.6 Erastin inhibits the intervening effect of FBR2 on ferroptosis

**FIGURE 6:**
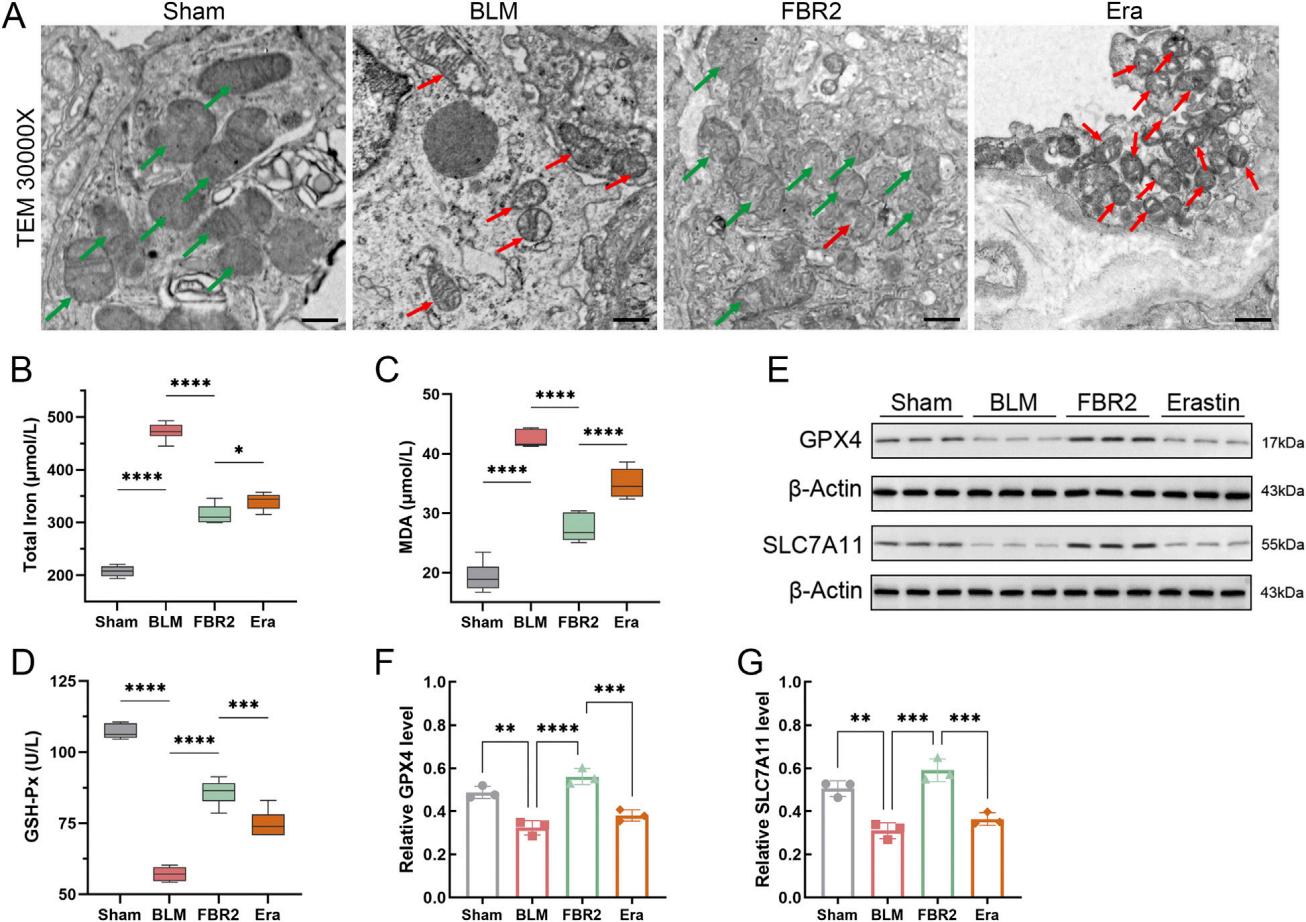
Erastin inhibits the intervening effect of FBR2 on ferroptosis. **(A)** Subcellular characteristic images of mouse lung tissue obtained by TEM, with red arrows indicating mitochondria with typical ferroptotic features such as shrunken morphology, increased membrane density, and reduced cristae, and green arrows pointing to relatively healthy mitochondria. Scale bar = 500 nm. **(B–D)** Levels of total iron, MDA, and GSH-Px in mouse lung tissue obtained using biochemical assay kits (n = 6). **(E–G)** Western blot and densitometric analysis of SLC7A11 and GPX4 protein expression in lung tissue. (n = 3) *p < 0.05; **p < 0.01; ***p < 0.001; ****p < 0.0001.

The small molecule Erastin is known to induce ferroptosis by modulating iron metabolism and inhibiting SLC7A11 (Dixon et al., 2012; Li et al., 2021). As depicted in Figure 6A, the mitochondria in the BLM group showed atrophy and increased membrane density compared to the healthy mitochondria in the Sham group, effects that were significantly mitigated by FBR2. However, in the Erastin group, which received TCM treatment, mitochondria displayed more pronounced “ferroptotic” characteristics. Assays for total iron and MDA revealed that FBR2’s beneficial effects on BLM-induced iron and lipid peroxide accumulation were negated in the Erastin-treated group (Figures 6B,C). Furthermore, Western blot and biochemical assays demonstrated that Erastin counteracted the upregulating effects of FBR2 on ferroptosis-protective molecules such as GSH-Px, GPX4, and SLC7A11 (Figures 6D–G). This evidence suggests that Erastin can neutralize the therapeutic effects of FBR2 on ferroptosis in BLM-treated mice.

### 3.7 FBR2 treats pulmonary injury and fibrosis in BLM-induced mice by targeting ferroptosis

**FIGURE 7:**
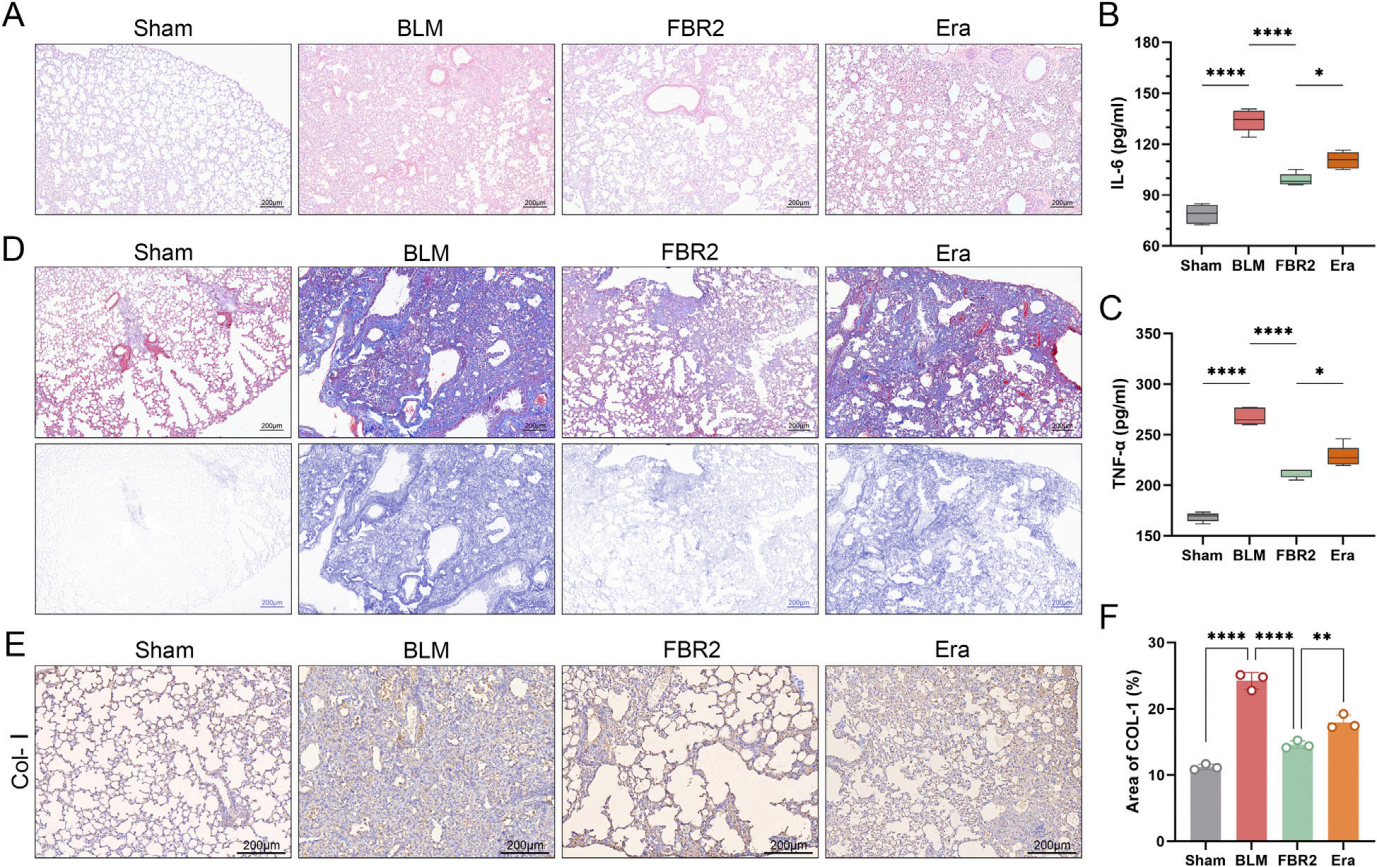
FBR2 treats pulmonary injury and fibrosis in BLM-induced mice by targeting ferroptosis. **(A)** Representative H&E staining images of mouse lung tissue. **(B, C)** Levels of IL-6 and TNF-α in mouse serum detected by ELISA. (n = 5) **(D)** Images of Masson’s trichrome staining of lung tissue, and distribution of collagen fibers obtained by color deconvolution analysis. **(E, F)** Expression of Col-I in lung tissue by immunohistochemistry and quantitative analysis of the positively stained area. (n = 3) **p < 0.01; ****p < 0.0001.

Considering that fibrosis levels in BLM-induced mice escalate with the activation of ferroptosis, we hypothesize that ferroptosis is a pivotal pathological process and a potential therapeutic target for fibrosis. H&E staining and ELISA measurements of IL-6 and TNF-α demonstrated that the addition of Erastin exacerbated lung injury in FBR2-treated mice (Figures 7A–C). Masson staining and IHC detection of Col-I reaffirmed that FBR2 can diminish collagen deposition; however, the findings in the Erastin group suggested that the ferroptosis inducer counteracted the anti-fibrotic effects of FBR2 (Figures 7D–F).

### 3.8 FBR2 improves ferroptosis and cellular senescence in BLM-induced mice via the SIRT3/p53 pathway

**FIGURE 8:**
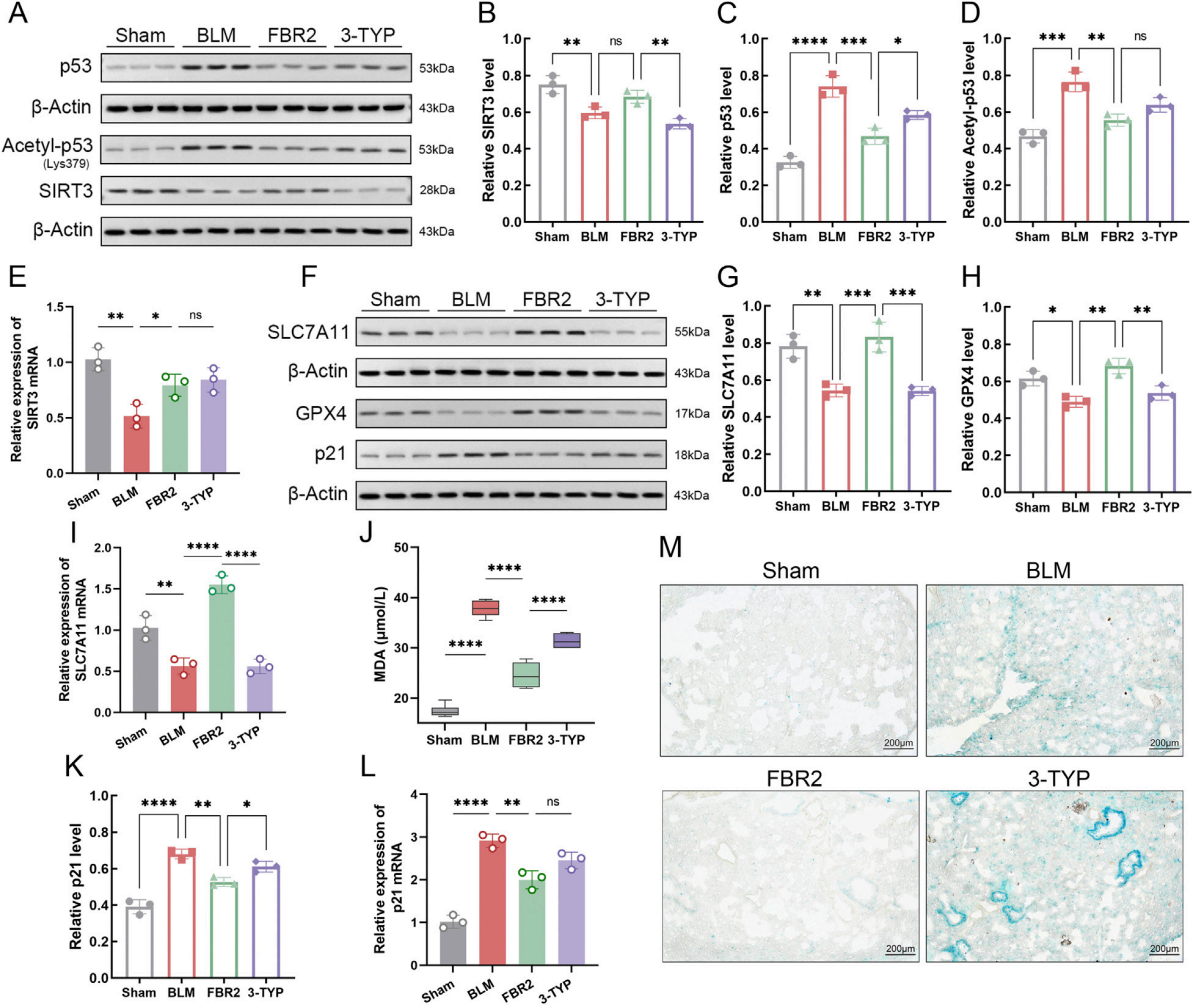
FBR2 improves ferroptosis and cellular senescence in BLM-induced mice via the SIRT3/p53 pathway. **(A)** Western blot and densitometric analysis of protein expression levels of SIRT3 **(B)**, p53 **(C)**, and Acetyl-p53 **(D)** in lung tissue. (n = 3) qRT-PCR detection of mRNA expression of SIRT3 **(E)**, SLC7A11 **(I)**, and p21 **(L)** in lung tissue. (n = 3) **(F)** Western blot and densitometric analysis of protein expression levels of SLC7A11 **(G)**, GPX4 **(H)**, and p21 **(K)**. (n = 3) **(J)** Detection of MDA expression level in lung tissue by assay kit. (n = 6) **(M)** Representative images of β-galactosidase staining in lung tissue. Scale bar = 200 μm *p < 0.05; **p < 0.01; ***p < 0.001; ****p < 0.0001; ns p > 0.05.

Studies have shown that SIRT3 can deacetylate p53 at K320 and K382 (equivalent to K379 in mice), promoting p53 degradation and inhibiting its transcriptional activity (Krummel et al., 2005; Nagasaka et al., 2022). We utilized the SIRT3 inhibitor 3-TYP to investigate the response of the SIRT3/p53 pathway to FBR2. Initially, we demonstrated that FBR2 has therapeutic effects on both the protein and mRNA suppression of SIRT3 induced by BLM. The addition of 3-TYP only counteracted the therapeutic effect of FBR2 on SIRT3 at the protein level, not the transcriptional level (Figures 8A,B,E). Subsequently, FBR2 significantly reduced the accumulation of p53 and acetylated p53 proteins induced by BLM, a trend reversed by 3-TYP. Notably, while the exacerbation of acetylated p53 by 3-TYP was not statistically significant, a discernible trend was observed (Figures 8A,C,D).

p53 can directly intervene in the SLC7A11/GPX4 pathway to promote ferroptosis (Liu and Gu, 2022). Detection of both protein and mRNA levels of SLC7A11 revealed that the therapeutic effects of FBR2 were reversed by the upstream inhibitor 3-TYP (Figures 8F,G,I). Further validation of the ameliorative effect of FBR2 on ferroptosis was provided by the assessment of GPX4 protein and MDA levels, which were antagonized by 3-TYP (Figures 8F,H,J).

Given the role of FBR2 in p53 and SIRT3, two proteins related to aging, we explored the significance of cellular senescence in the therapeutic process of FBR2. The results showed that p21, a direct target regulated by p53 for senescence (Li et al., 2012), was upregulated at both the protein and mRNA levels following BLM induction. FBR2 intervention corrected this upregulation, but 3-TYP reversed the therapeutic effect of FBR2 (Figures 8F,K,L). β-galactosidase staining, used to detect cellular senescence, confirmed that FBR2 aids in improving BLM-induced cellular senescence, an effect that was reversed by 3-TYP (Figure 8M). In conclusion, the therapeutic effects of FBR2 on ferroptosis and cellular senescence in BLM-induced pulmonary fibrosis mice are dependent on the SIRT3/p53 pathway.

### 3.9 FBR2 ameliorates pulmonary injury and fibrosis in BLM-induced mice through the SIRT3/p53 pathway

**FIGURE 9:**
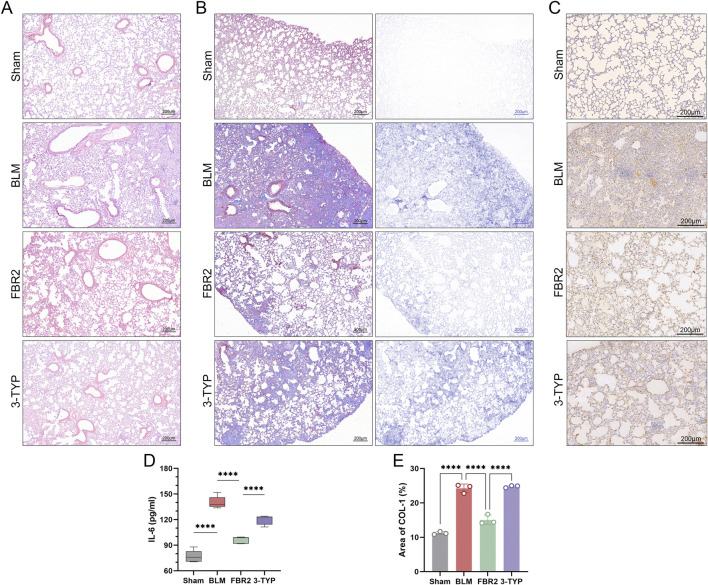
FBR2 ameliorates pulmonary injury and fibrosis in BLM-induced mice through the SIRT3/p53 pathway. **(A)** Representative H&E staining images of mouse lung tissue. **(B)** Images of Masson’s trichrome staining of lung tissue, and distribution of collagen fibers obtained by color deconvolution analysis. **(C)** Expression of Col-I in lung tissue by immunohistochemistry and **(E)** quantitative analysis of the positively stained area. (n = 3) **(D)** Detection of IL-6 level in mouse serum by ELISA. (n = 5) ***p < 0.001; ****p < 0.0001.

Ultimately, we explored the role of the SIRT3/p53 pathway in the therapeutic effects of FBR2 on pulmonary fibrosis. H&E staining and measurement of IL-6 levels revealed that the SIRT3 inhibitor 3-TYP counteracted the therapeutic effects of FBR2 on BLM-induced lung injury (Figures 9A,D). Masson staining and IHC detection of Col-I, demonstrated a significant reduction in collagen deposition in the FBR2 group, an effect that was negated by 3-TYP (Figures 9B,C,E). Thus, we confirmed that the protective effects of FBR2 against BLM-induced lung injury and fibrosis in mice are mediated by the SIRT3/p53 pathway.

## 4 Discussion

IPF, a common interstitial lung disease, is characterized by poor prognosis and limited response to conventional treatments. FBR2, a clinically validated TCM compound decoction, has demonstrated efficacy in treating IPF. Understanding the disease’s pathophysiology and the mechanisms underlying effective treatments is crucial for developing new drugs and optimizing therapeutic strategies. Increasing evidence implicates ferroptosis in lung injury and fibrosis (Jack et al., 1996; Ali et al., 2020). Studies have shown that ferroptosis can directly induce AT2 cell death, with lipid peroxide toxicity and iron ions triggering inflammatory and fibrotic pathways (Prockop, 1971). Cells counteract ferroptosis by activating defense mechanisms, with GPX4 converting lipid hydroperoxides into lipid alcohols, thereby reducing ROS levels and inhibiting ferroptosis. (Chen Xin et al., 2021). The activity of GPX4 relies on the antiport function of SLC7A11 for cystine/glutamate, which is essential for GSH cycle and synthesis. Thus, the SLC7A11/GPX4 axis is central to cellular defenses against ferroptosis. In IPF patients, reduced expression and activity of SLC7A11 and GPX4 have been observed, correlating with lung injury and fibrosis progression (Tsubouchi et al., 2019; Chen Tongshuai et al., 2021). Liu et al. (2024) confirmed that galanthamine alleviates BLM-induced pulmonary fibrosis in mice by enhancing the SLC7A11 pathway. Our findings indicate significant improvements in iron accumulation, lipid peroxidation damage, mitochondrial morphology, and the SLC7A11/GPX4 axis in FBR2-treated mice compared to the BLM group. The use of Erastin to reverse FBR2’s therapeutic effects further confirms the involvement of ferroptosis in the BLM-induced pulmonary fibrosis model in mice, suggesting that FBR2’s anti-fibrotic action is mediated through the inhibition of ferroptosis.

p53 is a transcription factor protein that primarily functions in the cell nucleus, regulating biological processes including cell cycle arrest, apoptosis, cellular senescence, and ferroptosis by binding to specific response elements. It has been reported that p53 can directly inhibit the expression of SLC7A11 at the transcriptional level, thereby promoting the occurrence of ferroptosis (Liu and Gu, 2022). The acetylation level of p53 is significantly elevated in response to various cellular stresses, a modification that is essential for augmenting p53’s stability, nuclear localization, and transcriptional activity (Ito et al., 2001). Our research indicates that FBR2 can ameliorate the accumulation and nuclear translocation of p53 protein induced by BLM. Moreover, the acetylation level of p53 is significantly reduced under FBR2 intervention. The inverse correlation between p53 protein and SIRT3 has been widely confirmed across various cells and tissues. Consequently, the significance of the SIRT3/p53 axis in processes such as ferroptosis, cellular aging, necrosis, and apoptosis is increasingly recognized (Li et al., 2023; Ye et al., 2023). Our findings reveal that the administration of a SIRT3 inhibitor not only restored the accumulation of p53 but also counteracted the therapeutic effects of FBR2 in BLM-induced mice. These results underscore the pivotal role of the SIRT3/p53 pathway in the modulation of ferroptosis by FBR2 and its therapeutic implications in pulmonary fibrosis.

Sirtuins are a family of deacetylases closely related to p53 function (Leeuwen and Lain, 2009, 53). Studies have shown that nuclear-expressed SIRT1 can deacetylate p53, thereby inhibiting its suppressive effect on SLC7A11 and protecting cardiomyocytes from ferroptosis (Jin et al., 2021). Given SIRT3’s role as a deacetylase, the modulation of post-translational modifications could be crucial in its regulation of p53’s transcriptional function. Su et al. found that the lack of SIRT3 can promote the cardiac fibrosis process through increased acetylation of p53 (Su et al., 2023). Xiong et al. have identified SIRT3’s direct deacetylation of p53 at lysines 320 and 382 using immunoprecipitation and colocalization assays (Xiong et al., 2018). Our colocalization studies indicate a potential direct interaction between SIRT3 and p53 in the presence of FBR2 treatment. Although our research suggests a trend where a SIRT3 inhibitor might counteract FBR2’s effect on reducing acetylated p53, this trend was not statistically significant. The specific mechanism by which FBR2 inhibits the acetylation level of p53 and its anti-ferroptotic transcriptional activity remains unclear. The role of SIRT3 upstream in this process and its precise function have yet to be determined. Furthermore, recent studies have suggested that SIRT3 could also enhance the ubiquitination of p53, leading to its degradation via the ubiquitin-proteasome pathway (Tang et al., 2020). This hypothesis presents an intriguing avenue for our upcoming research endeavors.

Considering the anti-aging roles of SIRT3 and p53, we examined cellular senescence phenotypes. As previously discussed, p53 can directly control the transcriptional expression of p21, regulating cell cycle arrest and the onset of cellular senescence. Our study also demonstrates a significant process of cellular senescence in the lung tissue of BLM-induced mice, and the anti-aging effect of FBR2 on these mice is mediated by the SIRT3/p53 pathway.

Our study encounters certain limitations. As previously mentioned, the precise manner in which SIRT3 affects the activity and levels of p53 protein in our treatment regimen requires further elucidation. To elucidate the details of protein post-translational modifications, additional methodologies such as co-immunoprecipitation, mass spectrometry, and genetic engineering will be required. As an early-stage exploratory study, we employed a single dose that was validated through clinical and *in vivo* experiments in our previous research to investigate the effects on ferroptosis and its upstream signaling pathways. Granular formulations of compound preparations are made and calculated according to strict standards and mature practices. The selection of this single dose complies with ethical considerations and ensures the efficient use of resources.

**FIGURE 10:**
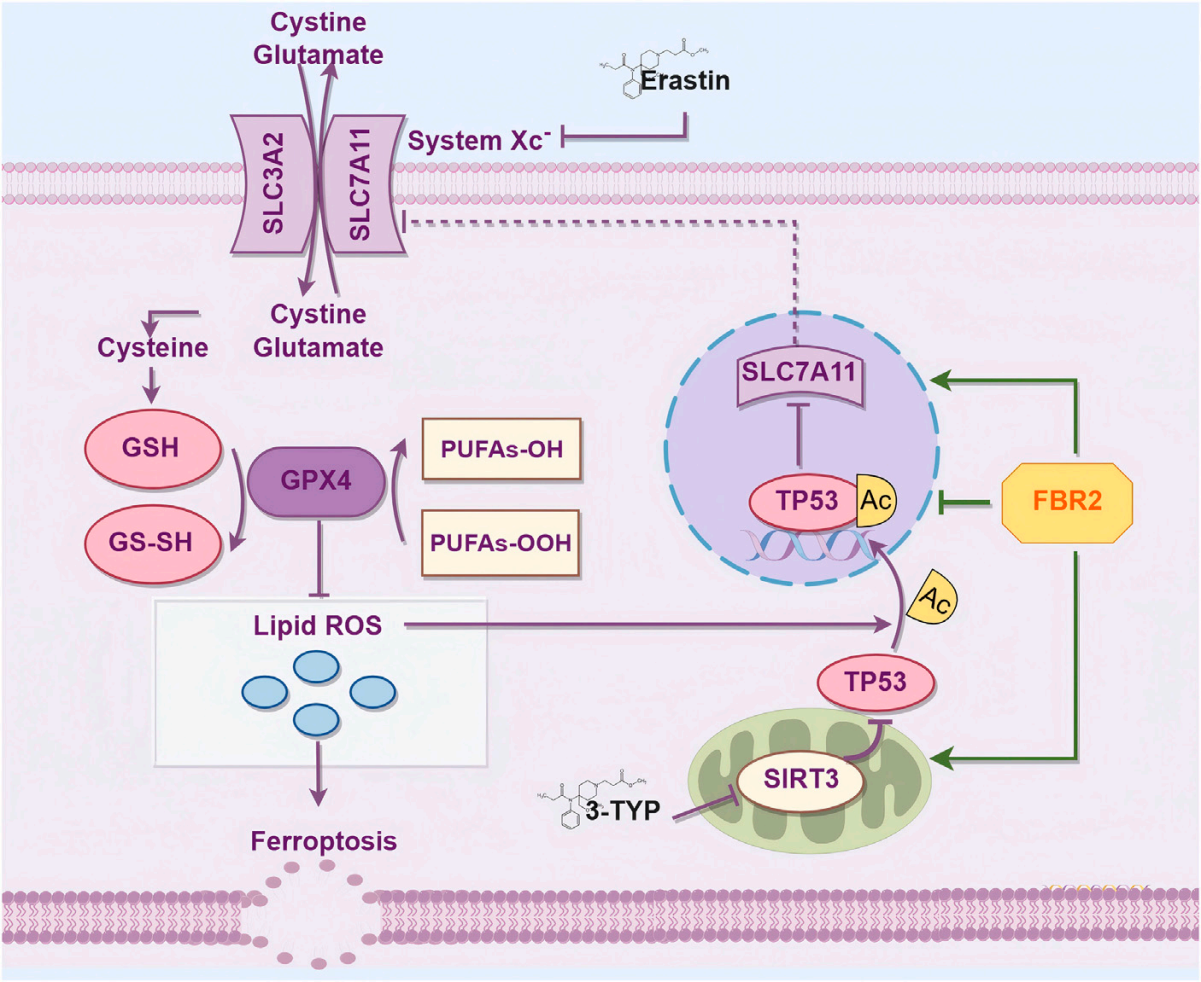
FBR2 Intervenes in Ferroptosis via the SIRT3/p53 Pathway to Treat BLM-Induced Pulmonary Fibrosis. This mechanism diagram was drawn using the Figdraw 2.0 tool (https://www.figdraw.com).

In summary, ferroptosis is a crucial therapeutic target for FBR2 in countering BLM-induced pulmonary fibrosis in mice. FBR2 can inhibit ferroptosis and cellular senescence induced by BLM through the SIRT3/p53 pathway, potentially involving alterations in the acetylation of p53, thereby treating pulmonary fibrosis (Figure 10). These findings provide novel evidence for the treatment of IPF by FBR2 and offer further insights into the pathogenesis of IPF.

## Data Availability

The original contributions presented in the study are included in the article/Supplementary Material, further inquiries can be directed to the corresponding authors.
