# Supplementary material for: FBR2 modulates ferroptosis via the SIRT3/p53 pathway to ameliorate pulmonary fibrosis
Source: Front Pharmacol. 2025 Feb 11;16:1509665. doi: 10.3389/fphar.2025.1509665 (PMC11850536; doi:10.3389/fphar.2025.1509665)
Supplement: Supplementary file 2 [file Table1.docx]

Supplementary Table 1 Main botanical drugs of FBR2

| **Chinese name** | **Botanical name** | **Family** | **Medicinal part** | **Place of origin of the botanical drug** |
| --- | --- | --- | --- | --- |
| Huang Qi | ***Astragalus mongholicus*** Bge. | Fabaceae | Root | Gansu Province, China |
| Hong Jing Tian | ***Rhodiola crenulata*** (Hook.f. & Thomson) H.Ohba | Crassulaceae | Rhizome and Root | Xizang Autonomous Region, China |
| Jin Yin Hua | ***Lonicera japonica*** Thunb. | Caprifoliaceae | Flower | Shandong Province, China |
| Huang Qin | ***Scutellaria baicalensis*** Georgi | Lamiaceae | Root | Shanxi Province, China |
| Dan Shen | ***Salvia miltiorrhiza*** Bge. | Lamiaceae | Rhizome and Root | Shandong Province, China |
| Gan Cao | ***Glycyrrhiza uralensis*** Fisch. ex DC. | Fabaceae | Rhizome and Root | Gansu Province, China |

Supplementary Table 2 Details on FBR2 Granule Dosage Conversion

| **Botanical drug** | **Crude Drug Dosage**  **(g)** | **Dosage equivalents**  **(g/g)** | **Granule (g)** |
| --- | --- | --- | --- |
| ***Astragalus mongholicus*** | 30 | 2.50 | 12 |
| ***Rhodiola crenulata*** | 30 | 3.00 | 10 |
| ***Lonicera japonica*** | 30 | 3.00 | 10 |
| ***Scutellaria baicalensis*** | 20 | 2.20 | 9.1 |
| ***Salvia miltiorrhiza*** | 20 | 2.00 | 10 |
| ***Glycyrrhiza uralensis*** | 10 | 3.00 | 3.33 |
| **Total** | 140 | 2.57 | 54.43 |

Supplementary Table 3 Identification and Testing of botanical drugs in FBR2 Granules

| **Project name** |  | **Result** |
| --- | --- | --- |
| *Lonicera japonica, Batch No.23009701, Test Date: 2023/3/30* | | |
| TLC Identification | No.1 | Complies with regulations |
| Characteristic | Color | Light yellow to yellow-brown |
|  | Odor | Slight fragrance |
|  | Taste | Bitter |
| Examination | Moisture | 3.3% |
|  | Granularity | 3.6% |
|  | Solubility | Complies with regulations |
|  | Heavy Metals and Harmful Elements | Complies with regulations |
| Characteristic Chromatogram | Chlorogenic Acid | 28.7mg/g |
|  | 3,5-Di-O-caffeoylquinic Acid  & 4,5-Di-O-caffeoylquinic Acid  & Chlorogenic Acid | 40.8mg/g |
|  | Luteolin-7-O-glucoside | 0.905mg/g |
| Extractables |  | 52.2% |
| Microbial and Control Bacteria | Aerobic Bacteria | 25cfu/g |
|  | Mold and Yeast | 20cfu/g |
|  | Escherichia coli | Not Detected/g |
| *Astragalus mongholicus, Batch No.24008591, Test Date: 2024/4/25* | | |
| TLC Identification | No.1 | Complies with regulations |
|  | No.2 | Complies with regulations |
| Characteristic | Color | Grayish-yellow to brownish-yellow |
|  | Odor | Slight |
|  | Taste | Slightly sweet, slightly bitter |
| Examination | Moisture | 0.046 |
|  | Granularity | 0.04 |
|  | Solubility | Complies with regulations |
|  | Heavy Metals and Harmful Elements | Lead: 0.06 mg/kg Cadmium: 0.008 mg/kg Arsenic: 0.2 mg/kg Mercury: Not Detected Copper: 2.7 mg/kg Organochlorines: Complies |
| Characteristic Chromatogram | Astragaloside | 1.75mg/g |
|  | Calycosin-7-O-glucoside | 0.75mg/g |
| Extractables |  | 28.7% |
| Microbial and Control Bacteria | Aerobic Bacteria | 15 cfu/g |
|  | Mold and Yeast | <10 cfu/g |
|  | Escherichia coli | Not Detected/g |
| *Rhodiola crenulata, Batch No.23022971, Test Date: 2023/08/19* | | |
| TLC Identification | No.1 | Complies with regulations |
| Characteristic | Color | Light Yellow-Brown to Red-Brown |
|  | Odor | Slight |
|  | Taste | Slightly Bitter and Astringent |
| Examination | Moisture | 3.3% |
|  | Granularity | 5% |
|  | Solubility | Complies with regulations |
|  | Heavy Metals and Harmful Elements | Complies with regulations |
| Characteristic Chromatogram | Salidroside | 10.8mg/g |
|  | Gallic Acid | 31.8mg/g |
| Extractables |  | 33.0% |
| Microbial and Control Bacteria | Aerobic Bacteria | <10 cfu/g |
|  | Mold and Yeast | <10 cfu/g |
|  | Escherichia coli | Not Detected/g |
| *Scutellaria baicalensis, Batch No.22009841, Test Date:2022-03-25* | | |
| TLC Identification | No.1 | Complies with regulations |
| Characteristic | Color | Yellow to Yellow-Brown |
|  | Odor | Slight |
|  | Taste | Bitter |
| Examination | Moisture | 3.6% |
|  | Granularity | 5.9% |
|  | Solubility | Complies with regulations |
|  | Heavy Metals and Harmful Elements | Complies with regulations |
| Characteristic Chromatogram | Baicalin | 155.1mg/g |
| Extractables |  | 24.5% |
| Microbial and Control Bacteria | Aerobic Bacteria | <10 cfu/g |
|  | Mold and Yeast | <10 cfu/g |
|  | Escherichia coli | Not Detected/g |
| *Scutellaria baicalensis, Batch No.22033521, Test Date:2022-10-25* | | |
| TLC Identification | No.1 | Complies with regulations |
| Characteristic | Color | Yellow-Brown to Brown |
|  | Odor | Slight |
|  | Taste | Slightly Bitter and Astringent |
| Examination | Moisture | 3.0% |
|  | Granularity | 5.8% |
|  | Solubility | Complies with regulations |
|  | Heavy Metals and Harmful Elements | Complies with regulations |
| Characteristic Chromatogram | Tanshinone IIA | 49.2mg/g |
| Extractables |  | 15.0% |
| Microbial and Control Bacteria | Aerobic Bacteria | <10 cfu/g |
|  | Mold and Yeast | <10 cfu/g |
|  | Escherichia coli | Not Detected/g |
| *Glycyrrhiza uralensis, Batch No.22036031, Test Date:2022-09-15* | | |
| TLC Identification | No.1 | Complies with regulations |
| Characteristic | Color | Yellow to Yellow-Brown |
|  | Odor | Slight |
|  | Taste | Sweet and Distinctive |
| Examination | Moisture | 3.7% |
|  | Granularity | 7.0% |
|  | Solubility | Complies with regulations |
|  | Heavy Metals and Harmful Elements | Complies with regulations |
| Characteristic Chromatogram | Glycyrrhizin | 16.4mg/g |
|  | Glycyrrhizic Acid | 39.9mg/g |
| Extractables |  | 40.6% |
| Microbial and Control Bacteria | Aerobic Bacteria | <10 cfu/g |
|  | Mold and Yeast | <10 cfu/g |
|  | Escherichia coli | Not Detected/g |

Supplementary Table 4 LC mobile phase conditions

| **Time**  **(min)** | **Flow rate**  **(μL/min)** | **Gradient** | **B%**  **Acetonitrile** | **A%**  **Fomic acid** |
| --- | --- | --- | --- | --- |
| 0-2 | 300 | - | 5 | 95 |
| 2-6 | 300 | Linear gradient | 30 | 70 |
| 6-7 | 300 | - | 30 | 70 |
| 7-12 | 300 | Linear gradient | 78 | 22 |
| 12-14 | 300 | - | 78 | 22 |
| 14-17 | 300 | Linear gradient | 95 | 5 |
| 17-20 | 300 | - | 95 | 5 |
| 20-21 | 300 | Linear gradient | 5 | 95 |
| 21-25 | 300 | - | 5 | 95 |

Supplementary Table 5 Detailed List of Identified Metabolites in FBR2

| **NO.** | **Main metabolites** | **Formula** | **Error [ppm]** | **RT [min]** | **Peak area** | **Concentration（μg/mL）** |
| --- | --- | --- | --- | --- | --- | --- |
| 1 | Baicalin | C21H18O11 | -1.25 | 7.769 | 18223786758 | 953.1748776 |
| 2 | Wogonoside | C22H20O11 | -0.99 | 9.028 | 7491954736 | 391.8583516 |
| 3 | Pelargonidin | C15H10O5 | -1.32 | 10.13 | 5805200076 | 303.634527 |
| 4 | 2-Pyrrolidinecarboxylic acid | C5H9NO2 | 1.67 | 0.809 | 5262239021 | 275.2355535 |
| 5 | Oroxylin A-7-O-β-D-glucuronide | C22H20O11 | -1.06 | 8.685 | 4245289444 | 222.045138 |
| 6 | Choline | C5H13NO | 2.96 | 0.765 | 4218627490 | 220.6506142 |
| 7 | Oroxylin A | C16H12O5 | -1 | 11.183 | 3590731251 | 187.8092005 |
| 8 | Chlorogenic acid | C16H18O9 | -1.23 | 5.24 | 3047864376 | 159.415153 |
| 9 | Apigenin 7-O-glucuronide | C21H18O11 | -0.99 | 8.36 | 2954122677 | 154.5120978 |
| 10 | Chrysosplenetin B | C19H18O8 | -1.13 | 11.274 | 2511548280 | 131.3637366 |
| 11 | DL-Arginine | C6H14N4O2 | -0.51 | 0.773 | 2478631931 | 129.6420837 |
| 12 | 7-Hydroxycoumarine | C9H6O3 | -0.61 | 5.238 | 2409953918 | 126.0499566 |
| 13 | Liquiritigenin | C15H12O4 | -1.07 | 6.703 | 2058597487 | 107.6726497 |
| 14 | 1-[(3-Carboxypropyl)amino]-1-deoxy-β-D-fructofuranose | C10H19NO7 | -1.36 | 0.788 | 1968345349 | 102.9521121 |
| 15 | Wogonin | C16H12O5 | -1.09 | 11.408 | 1704551593 | 89.15467337 |
| 16 | Caffeic acid | C9H8O4 | -0.96 | 5.361 | 1533655751 | 80.21615664 |
| 17 | L-Pyroglutamic acid | C5H7NO3 | 0.69 | 1.158 | 1503687372 | 78.64869391 |
| 18 | 2,4-Xylidine | C8H11N | 1.27 | 5.307 | 1232044288 | 64.44070485 |
| 19 | Mirificin | C26H28O13 | -0.39 | 6.739 | 1164011648 | 60.88233335 |
| 20 | Calycosin | C16H12O5 | -1.4 | 9.015 | 1160566173 | 60.70212161 |
| 21 | Diammonium glycyrrhizinate | C42H62O16 | -0.87 | 10.073 | 1123570637 | 58.76711131 |
| 22 | Acetylvanillin | C10H10O4 | -0.97 | 6.371 | 1113030173 | 58.21580403 |
| 23 | Cryptotanshinone | C19H20O3 | -1.13 | 13.408 | 1090597044 | 57.04246419 |
| 24 | 18 β-Glycyrrhetintic Acid | C30H46O4 | -1.17 | 10.073 | 1089444680 | 56.98219111 |
| 25 | Trigonelline HCl | C7H7NO2 | -0.53 | 0.838 | 1077914109 | 56.37909743 |
| 26 | Coumestrol | C15H8O5 | -1.35 | 10.058 | 1035188533 | 54.14438371 |
| 27 | Guaiacol | C7H8O2 | 2.15 | 5.361 | 1032054469 | 53.98046003 |
| 28 | Phenylglyoxylic acid | C8H6O3 | -0.24 | 6.119 | 1009244440 | 52.78740683 |
| 29 | 3,5-Dicaffeoylquinic acid | C25H24O12 | -0.7 | 7.3 | 1002840292 | 52.45244498 |
| 30 | 3,4-Dihydroxyphenylacetic acid | C8H8O4 | -0.2 | 5.207 | 976700741.9 | 51.08524493 |
| 31 | Ononin | C22H22O9 | -0.74 | 7.847 | 946765526.5 | 49.51951682 |
| 32 | Iminodimethanethiol | C2H7NS2 | -3.81 | 0.683 | 911498928.9 | 47.67493669 |
| 33 | Sweroside | C16H22O9 | -1.24 | 5.83 | 866520787.3 | 45.32240508 |
| 34 | syringetin | C17H14O8 | -1.27 | 8.25 | 845883143.2 | 44.24297607 |
| 35 | Genistin | C21H20O10 | -1.2 | 7.789 | 845630364.8 | 44.22975478 |
| 36 | Cantharidin | C10H12O4 | -0.37 | 5.83 | 793790774.5 | 41.51834272 |
| 37 | Daidzein-7-O-glucuronide | C21H18O10 | -0.67 | 8.759 | 791796365.3 | 41.41402737 |
| 38 | Tanshinone IIA | C19H18O3 | -0.92 | 14.65 | 778419069.5 | 40.71434281 |
| 39 | Baicalein | C15H10O5 | -4.44 | 7.772 | 712349759.5 | 37.25866111 |
| 40 | Cryptochlorogenic acid | C16H18O9 | -1.23 | 5.39 | 706930607.3 | 36.97521838 |
| 41 | Calycosin-7-O-β-D-glucoside | C22H22O10 | -0.95 | 6.546 | 690114998.5 | 36.09569668 |
| 42 | Pectolinarigenin | C17H14O6 | -1.34 | 11.299 | 682746320.8 | 35.7102862 |
| 43 | L-Glutamic acid | C5H9NO4 | -1.12 | 0.785 | 675208771 | 35.31604305 |
| 44 | 6-O-Methylscutellarin | C22H20O12 | -0.71 | 8.463 | 611447893.3 | 31.98110133 |
| 45 | Verbenalin | C17H24O10 | -1.07 | 6.375 | 607144586.8 | 31.75602167 |
| 46 | Asparagine | C4H8N2O3 | -0.26 | 0.783 | 584519971 | 30.57266633 |
| 47 | Formononetin | C16H12O4 | -0.8 | 10.513 | 545491042.6 | 28.53130168 |
| 48 | Adenosine | C10H13N5O4 | -0.85 | 1.202 | 535313644.8 | 27.99898422 |
| 49 | DL-Stachydrine | C7H13NO2 | 0.03 | 0.854 | 526801187.5 | 27.55374962 |
| 50 | Sinapic acid | C11H12O5 | -0.78 | 6.118 | 511361501.2 | 26.74619401 |
| 51 | L-Canavanine | C5H12N4O3 | -0.88 | 0.78 | 481125231.6 | 25.1647196 |
| 52 | Adenine | C5H5N5 | -0.09 | 1.134 | 432945444.4 | 22.64472946 |
| 53 | Salidroside | C14H20O7 | -1.07 | 4.666 | 407958452.6 | 21.33781268 |
| 54 | Isochlorogenic acid B | C25H24O12 | -0.28 | 6.955 | 392204540.1 | 20.51382182 |
| 55 | Lithospermic acid | C27H22O12 | -0.88 | 7.807 | 390267158.9 | 20.41248926 |
| 56 | L-Leucine | C6H13NO2 | 0.71 | 1.369 | 374511572.1 | 19.58841083 |
| 57 | Quercitrin | C21H20O11 | -1.28 | 8.25 | 370799631.1 | 19.39426189 |
| 58 | Isochlorogenic acid C | C25H24O12 | -0.42 | 7.163 | 357228695.2 | 18.6844492 |
| 59 | Emodin | C15H10O5 | -1.05 | 11.374 | 348922784.9 | 18.25001781 |
| 60 | Isoliquiritin | C21H22O9 | -1.51 | 7.745 | 334677160.1 | 17.50491626 |
| 61 | 4-Methylumbelliferone | C10H8O3 | -0.61 | 6.374 | 318668139.7 | 16.66758227 |
| 62 | L-Phenylalanine | C9H11NO2 | -0.03 | 2.222 | 304130899.7 | 15.90722812 |
| 63 | Icaritin | C21H20O6 | -0.81 | 11.304 | 294741774.4 | 15.41614037 |
| 64 | L-Threonine | C4H9NO3 | 1.11 | 0.774 | 283048230.7 | 14.80452258 |
| 65 | D-glucosamine | C6H13NO5 | -0.12 | 0.719 | 279324466.6 | 14.60975525 |
| 66 | p-Coumaric acid | C9H8O3 | 0.15 | 1.227 | 261654463.1 | 13.68554539 |
| 67 | Dihydrotanshinone I | C18H14O3 | -0.85 | 12.483 | 257073018.9 | 13.44591805 |
| 68 | Kaempferol | C15H10O6 | -0.87 | 9.912 | 256253559.2 | 13.40305712 |
| 69 | Swertiamarin | C16H22O10 | -1.15 | 5.362 | 240502437.2 | 12.57921222 |
| 70 | Isoliquiritigenin | C15H12O4 | -1.06 | 8.84 | 234913917 | 12.2869109 |
| 71 | Astragalin | C21H20O11 | -0.65 | 6.736 | 232791652.4 | 12.17590821 |
| 72 | Chrysin | C15H10O4 | -0.85 | 11.215 | 207586554.4 | 10.85758362 |
| 73 | Amygdalin | C20H27NO11 | -0.71 | 6.638 | 206488323.5 | 10.80014188 |
| 74 | D-(+)-Pipecolinic acid | C6H11NO2 | 0.43 | 1.129 | 196734203.4 | 10.28996348 |
| 75 | 5-Hydroxymethylfurfural | C6H6O3 | 0.74 | 5.83 | 188335529.2 | 9.850680178 |
| 76 | L-Valine | C5H11NO2 | 1.05 | 0.815 | 186896209.1 | 9.775398141 |
| 77 | Scutellarein | C15H10O6 | -1.41 | 7.763 | 180252579.6 | 9.4279105 |
| 78 | Glycitin | C22H22O10 | -1.83 | 8.629 | 177506267 | 9.284267675 |
| 79 | Eupatilin | C18H16O7 | -0.74 | 11.592 | 174817878.1 | 9.143654487 |
| 80 | Alternariolmethylether | C15H12O5 | -1.26 | 8.251 | 169956142.3 | 8.889366809 |
| 81 | trans-3-Indoleacrylic acid | C11H9NO2 | -0.55 | 4.555 | 165599105.9 | 8.66147687 |
| 82 | Lysionotin | C18H16O7 | -0.65 | 11.04 | 163578550.7 | 8.555793981 |
| 83 | Bis(4-ethylbenzylidene)sorbitol | C24H30O6 | -1.11 | 11.979 | 161998427.9 | 8.473147415 |
| 84 | Phthaldialdehyde | C8H6O2 | 0.12 | 5.24 | 160870659.6 | 8.414160751 |
| 85 | Puerarin | C21H20O9 | -1 | 7.845 | 160312531.2 | 8.384968468 |
| 86 | Scutellarin | C21H18O12 | -0.66 | 6.716 | 158555802.5 | 8.293084728 |
| 87 | Secoxyloganin | C17H24O11 | -0.62 | 4.949 | 152495278.2 | 7.976095752 |
| 88 | Alisol C 23-acetate | C32H48O6 | 0.1 | 9.367 | 151796706.8 | 7.939557752 |
| 89 | Iristectorigenin B | C17H14O7 | -1.07 | 10.185 | 149654715.1 | 7.827523259 |
| 90 | Protocatechualdehyde | C7H6O3 | 0.03 | 5.044 | 143627337.1 | 7.512267966 |
| 91 | Quillaic acid | C30H46O5 | -0.44 | 9.436 | 143130990.8 | 7.486307126 |
| 92 | Kaempferol 3-glucorhamnoside | C27H30O15 | -0.43 | 6.628 | 142952417.8 | 7.476967064 |
| 93 | Hispidulin | C16H12O6 | -0.72 | 10.279 | 141741343.4 | 7.413623164 |
| 94 | Hyperoside | C21H20O12 | -0.76 | 6.721 | 127643919.4 | 6.676273098 |
| 95 | Luteolin | C15H10O6 | -1.15 | 7.973 | 124292016.5 | 6.500955548 |
| 96 | Acetophenone | C8H8O | 1.48 | 4.665 | 119302044.2 | 6.239960603 |
| 97 | Cinnamic acid | C9H8O2 | -0.11 | 6.376 | 115081355.1 | 6.019202159 |
| 98 | Guanosine | C10H13N5O5 | -0.93 | 1.296 | 106705759.7 | 5.581125967 |
| 99 | Diosmetin | C16H12O6 | -1.09 | 7.414 | 102303317 | 5.350861101 |
| 100 | Mulberrin | C25H26O6 | -0.97 | 13.26 | 102251810.2 | 5.348167097 |
